# Natural Killer Cell Mediated Missing-Self Recognition Can Protect Mice from Primary Chronic Myeloid Leukemia *In Vivo*

**DOI:** 10.1371/journal.pone.0027639

**Published:** 2011-11-23

**Authors:** Mika Kijima, Noémie Gardiol, Werner Held

**Affiliations:** Ludwig Center for Cancer Research of the University of Lausanne, Epalinges, Switzerland; University of Cape Town, South Africa

## Abstract

**Background:**

Natural Killer (NK) cells are thought to protect from residual leukemic cells in patients receiving stem cell transplantation. However, multiple retrospective analyses of patient data have yielded conflicting conclusions regarding a putative role of NK cells and the essential NK cell recognition events mediating a protective effect against leukemia. Further, a NK cell mediated protective effect against primary leukemia *in vivo* has not been shown directly.

**Methodology/Principal Findings:**

Here we addressed whether NK cells have the potential to control chronic myeloid leukemia (CML) arising based on the transplantation of BCR-ABL1 oncogene expressing primary bone marrow precursor cells into lethally irradiated recipient mice. These analyses identified missing-self recognition as the only NK cell-mediated recognition strategy, which is able to significantly protect from the development of CML disease *in vivo*.

**Conclusion:**

Our data provide a proof of principle that NK cells can control primary leukemic cells *in vivo*. Since the presence of NK cells reduced the abundance of leukemia propagating cancer stem cells, the data raise the possibility that NK cell recognition has the potential to cure CML, which may be difficult using small molecule BCR-ABL1 inhibitors. Finally, our findings validate approaches to treat leukemia using antibody-based blockade of self-specific inhibitory MHC class I receptors.

## Introduction

Chronic myeloid leukemia (CML) is a myeloproliferative disorder characterized by a reciprocal translocation between chromosome 9 and 22, the so-called Philadelphia (Ph) chromosome. This translocation juxtaposes the genes encoding the ABL1 tyrosine kinase and BCR (Breakpoint cluster region), resulting in a BCR-ABL1 fusion protein with constitutive tyrosine kinase activity. This activity is critically involved in the initial chronic phase of CML disease and the subsequent disease progression. Indeed, the BCR-ABL1 inhibitor imatinib has become the standard therapy in newly diagnosed CML patients. Based on multiple clinical studies, a majority of patients (52–69%) achieve a complete cytogenic response (i.e. no Ph+ metaphases in 20/20 cells) but only a minority of patients (12–40%) achieve a major molecular response (i.e. a 3-log reduction in BCR-ABL1 mRNA) by 12 months of treatment [1]. Nilotinib and desatinib are second-generation inhibitors that exhibit considerably higher activity against BCR-ABL1 and that show further increased response rates [1]. Despite the impressive ability to control disease, there are CML patients that do not respond to BCR-ABL1 inhibitors or in which the disease progresses, some times based on mutations in BCR-ABL1. Finally, recurrence has been observed in a significant fraction of patients when BCR-ABL1 inhibitor treatment is discontinued [2], suggesting that leukemia initiating cells may persist and be refractory to inhibitor treatment. Thus additional treatment options, which are able to target leukemia-propagating cells, are needed to treat certain CML patients.

Haematopoietic stem cell transplantation has the potential to cure CML [3]. This is in part due to immune cells present in the graft and/or developing from grafted stem cells, which mediate a graft versus leukemia (GvL) effect to eliminate residual leukemic cells. Unrelated HLA-matched and partially HLA-mismatched transplants may contain T cells, which recognize minor histocompatibility antigens or HLA molecules on residual leukemic cells, respectively. However, such T cell recognition bears the significant risk of graft versus host disease (GvHD), a life threatening complication of (partially) HLA mismatched stem cell transplantation, in which donor-derived T cells attack non-haematopoietic, healthy tissues of the recipient. A partial HLA mismatch can also be recognized by NK cells and it has been suggested that alloreactive NK cells can prevent leukemia relapse following stem cell transplantation [4]. In contrast to T cells, NK cells do not seem to cause GvHD [5], [6].

NK cells can react to allogeneic cells based on various recognition events. First some NK cells can be activated using receptors, which are specific for allogeneic MHC-I [7], [8]. In addition, many NK cells express inhibitory receptors specific for MHC-I [9], [10]. MHC-I receptors counteract NK cell activation by receptors specific for ligands that are constitutively expressed on healthy host cells. This dual receptor system allows the killing of diseased host cells, which display aberrantly low levels of MHC-I molecules (missing-self recognition) [11]. Since inhibitory MHC-I receptors (KIR (Killer Immunoglobulin-like Receptors) in human and Ly49 family receptors in mice) do not recognize all MHC-I alleles, the dual receptor system can confer reactivity to allogeneic cells that express the wrong MHC-I (KIR ligand mismatch). NK cell alloreactivity is further dependent on NK cell education [12] i.e. activation receptors on NK cells, which express a KIR/Ly49 specific for self-MHC-I respond more efficiently to stimulation [13], [14], [15], [16]. Consequently, NK cell alloreactivity depends on the expression of a KIR/Ly49 and its MHC-I ligand in the donor (for education) and the absence of MHC-I ligand in the recipient (for the relieve from inhibition). Conversely, the activation receptors on NK cells that do not express a KIR/Ly49 specific for self-MHC-I respond poorly stimulation [13], [14], [15], [16]. However, the function of these activation receptors can improve when these uneducated NK cells are exposed to inflammatory cytokines [14], [17]. Consequently, it is possible that uneducated NK cells acquire reactivity due to the peculiar inflammatory environment during stem cell transplantation [18]. In addition to NK cell alloreactivity, there is evidence that the upregulation of stress induced self ligands, such as those engaging the NKG2D activation receptor [19], represents an important NK cell recognition strategy for transformed cells [20]. The importance of this pathway in the context of GvL is not known.

A role of NK cells to protect from leukemia has been inferred from the retrospective analysis of data from patients receiving unrelated HLA matched or partially HLA mismatched hematopoietic stem cell transplantation. A reduced relapse and improved disease free survival of leukemia patients receiving partially mismatched transplants has been attributed to NK cell alloreactivity mediated by the absence of KIR ligand in the recipient (KIR-ligand mismatch i.e. missing-self recognition) [4]. However, certain studies have either found no beneficial effect of KIR ligand mismatch or reported even worse outcomes following partially HLA mismatched stem cell transplantation [21], [22]. Moreover, other studies have proposed protective roles for NK cell recognition of allogenic HLA using activating KIR [23], [24] or they suggested a role for uneducated NK cells [25], [26], [27] (for review see [28], [29]). Some of these discrepancies may be due to distinct conditioning regimens, differences in the preparation, the source and the dose of the transplanted stem cells and/or the fact that the cohorts included patients with distinct hematological malignancies that are based on different primary genetic lesions.

Despite some of the above correlations, it has not been shown directly that NK cells control primary leukemic cells arising *in vivo* and, if so, it is not clear which NK cell recognition strategy would be most effective against leukemic cells. Finally it is not known whether NK cells can target leukemia initiating stem cells and thus whether NK cells have the potential to contribute to curing leukemia.

Here we have addressed whether NK cells do have the potential to control primary CML *in vivo*. To this end, we used a well-established mouse model, in which bone marrow (BM) precursor cells are transduced with the BCR-ABL1 oncogene. Infected BM cells are transplanted into lethally irradiated recipient mice, which develop a fatal CML disease [30], [31]. This disease model was combined with the classical model of BM graft rejection mediated by host NK cells [32], which allowed us to determine whether there was a difference in the efficacy of NK cells against normal and leukemic cells. Our analyses reveal that NK cell-mediated missing-self recognition, but none of the other NK cell recognition strategies, significantly impacts the outgrowth of CML cells *in vivo*. The NK cell mediated effect is based at least in part on the targeting of leukemia initiating stem cells, which suggests that NK cell recognition may contribute to cure CML disease.

## Results

### Lack of NK cell recognition of MHC-I matched BCR-ABL1+ myeloid cells *in vivo*

To determine whether NK cells can protect against primary BCR-ABL1+ cells *in vivo*, BM progenitor cells were infected with a retrovirus encoding the BCR-ABL1 (p210) oncogene plus green fluorescent protein (GFP) or with a control retrovirus expressing only GFP. Transduced BM precursor cells (usually around 10% GFP+) were transplanted into lethally irradiated recipient mice. Polymorphisms in the CD45 molecule were utilized to discriminate hematopoietic cells of donor (CD45.1) and recipient (CD45.2) origin.

**Figure 1:**
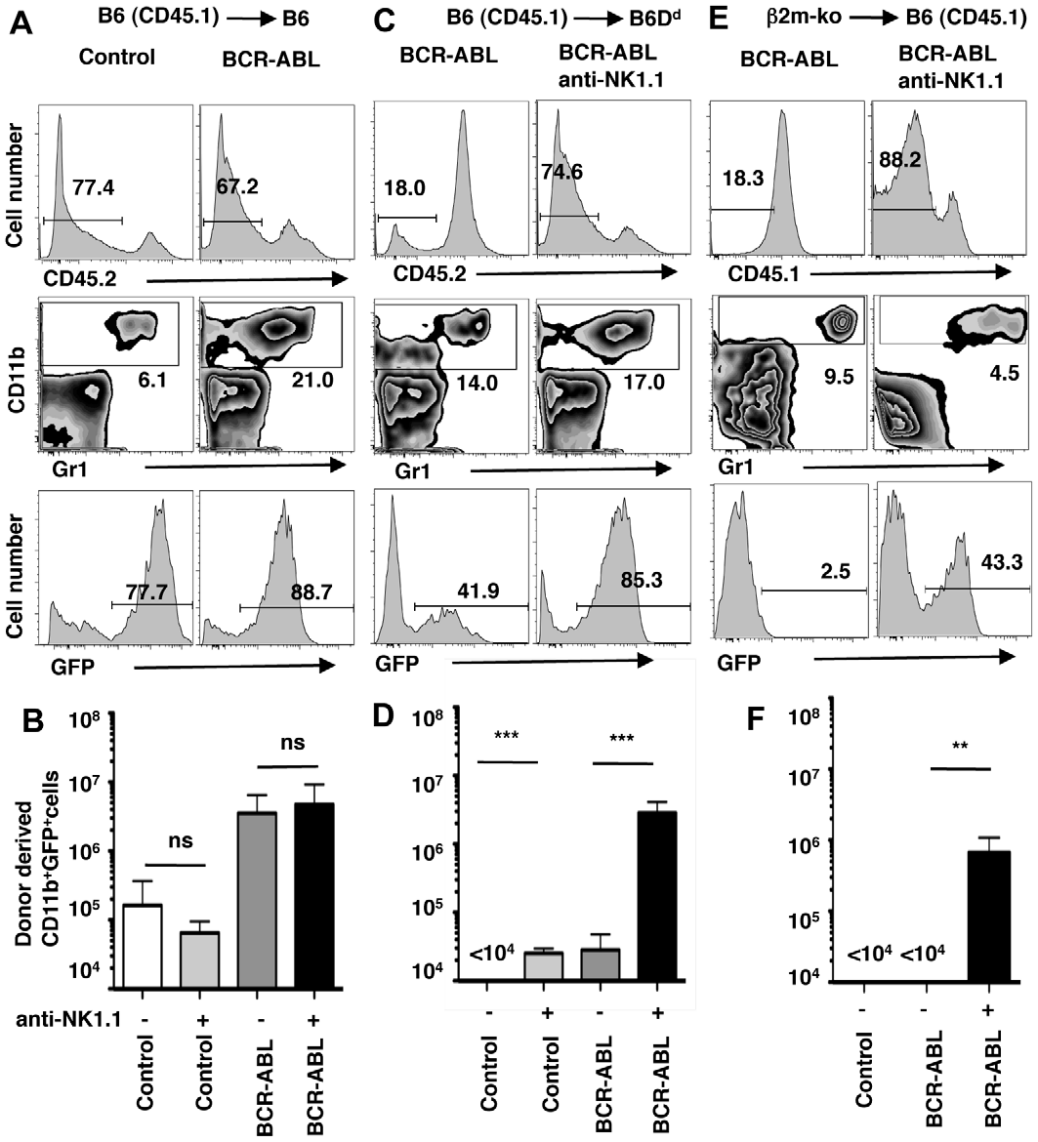
NK cell mediated missing-self recognition reduces the expansion of BCR-ABL1+ myeloid cells *in vivo*. BM cells from 5-FU treated B6 mice (H-2^b^, CD45.1) were transduced with retroviral vectors encoding GFP (Control) or BCR-ABL1 plus GFP (BCR-ABL1) followed by transplantation into lethally irradiated, MHC-I matched B6 (H-2^b^, CD45.2) mice (**A, B**) or MHC-I-different B6 D^d^ mice (H-2^b^D^d^, CD45.2) (**C, D**). In addition, BM cells from 5-FU treated β2m-deficient (β2m-ko) mice (MHC-I^low^, CD45.2) were transduced with retroviral vectors encoding BCR-ABL1 plus GFP followed by transplantation into B6 (H-2^b^, CD45.1) mice (**E, F**). Some recipient mice had been depleted of NK1.1+ cells by the injection of mAb PK136 (anti-NK1.1). Histograms show the identification of donor-derived cells at day 8 after transplantation (upper panel). Donor-derived cells were further analyzed for the presence of myeloid cells (CD11b+)(middle panel) and GFP expression in donor-derived myeloid cells (lower panel). Numbers indicate the percentage of cells in the respective gate. Bar graphs show the mean absolute number (±SD) of donor-derived myeloid cells expressing GFP (either control or BCR-ABL1) in recipient spleens at day 8 after transplantation. Donor-recipient mouse strain combinations were (**B**) B6 (H-2^b^, CD45.1)->B6 (H-2^b^, CD45.2) (MHC-I identical) and (**D**) B6 ((H-2^b^, CD45.1) into B6D^d^ (H-2^b^D^d^, CD45.2) (MHC-I different, partial missing-self) and (**F**) β2m-ko (MHC-1^low^, CD45.2)−>B6 (H-2^b^, CD45.1) (MHC-I deficient, complete missing-self). The significance of the difference between indicated data sets is depicted as *** p<0.001, ** p<0.01 and ns not significantly different *p*>0.05.

To address whether NK cells protect against MHC-I matched BCR-ABL1+ cells, we estimated the abundance of GFP+ myeloid cells (CD11b+) in recipient spleens 8 days (d8) after transplantation (**Fig. 1**). B6 (H-2^b^)-derived BM precursors transduced with the control virus yielded low numbers of GFP+ myeloid cells in spleens of MHC-I matched B6 recipients (H-2^b^) (10^5^ cells/spleen) (**Fig. 1A, B**). Transduction with the BCR-ABL1 virus resulted in significantly elevated numbers of GFP+ CD11b+ cells (5×10^6^ cells) (**Fig. 1A, B**), confirming that BCR-ABL1 expression induces an expansion of myeloid cells. The depletion of NK1.1+ cells prior to BM transplantation did not impact the abundance of BCR-ABL1 expressing CD11b+ cells (**Fig. 1B**), indicating that NK cells do not react to MHC-I-matched BCR-ABL1 transformed cells.

### NK cell mediated missing-self recognition reduces the expansion of BCR-ABL1+ myeloid cells

We next addressed whether a partial MHC-I mismatch, which results in NK cell mediated missing-self recognition, impacts the expansion of BCR-ABL1 expressing cells. Control infected B6 BM precursors yield myeloid cells in B6 hosts (10^5^ cells) (**Fig. 1B**) but not in B6 hosts expressing a H-2D^d^ transgene (H-2^b^D^d^)(<10^4^ cells) (**Fig. 1D**), illustrating NK cell mediated rejection of normal BM grafts based on missing-self recognition [33], [34]. The transplantation of BCR-ABL1 B6 BM progenitors into B6D^d^ hosts yielded a large number of BCR-ABL1+ myeloid cells (>10^6^ cells) when NK1.1+ cells had been depleted (**Fig. 1C, D**). When NK cells were present, the number of BCR-ABL1+-CD11b+ cells was approximately 30 fold lower (**Fig. 1C, D**). Thus a partial MHC-I mismatch significantly diminished the expansion of BCR-ABL1+ cells *in vivo* due to the presence of NK cells. This is likely mediated by NK cells expressing inhibitory Ly49A+ and Ly49G2+ receptors, which are inhibited by H-2D^d^ but not H-2^b^ molecules [35].

We next tested whether complete MHC-I deficiency further accentuated the protective effect. Indeed, the abundance of BCR-ABL1+ myeloid cells that developed from β2m-deficient (β2m-ko, MHC-I^low^) BM precursors was reduced 500 fold in the presence of NK cells in B6 recipient mice (H-2^b^) (**Fig. 1E, F**). We conclude that NK cell mediated missing-self recognition can efficiently control myeloid expansion driven by the BCR-ABL1 oncogene *in vivo*. The improved protective effect is likely due to a higher number of mismatches between inhibitory receptors and MHC-I ligands as observed for the NK cell mediated rejection of MHC-I-different normal cells [36].

### NK cell mediated “non-self” or “induced-self” recognition fails to control BCR-ABL1+ cells

**Figure 2:**
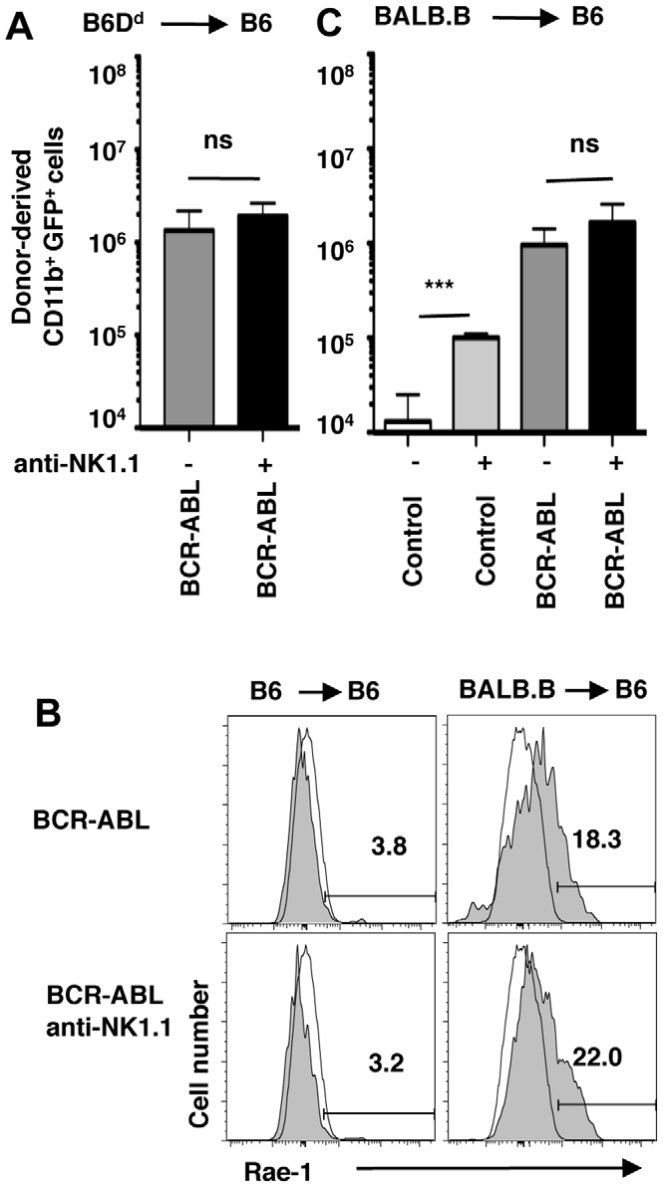
NK cell mediated non-self or induced-self recognition does not impact the expansion of BCR-ABL1+ cells. (**A**) The bar graph shows the mean absolute number (±SD) of B6 D^d^ (H-2^b^D^d^, CD45.1)-derived myeloid cells expressing BCR-ABL1 (GFP) in B6 (H-2^b^, CD45.2) recipients at day 8 after transplantation (MHC-I different, non-self recognition). (**B**) The expression of Rae-1 NKG2D ligands was determined on B6 (left) or BALB.B (right)-derived myeloid cells (CD11b+) expressing BCR-ABL1 on day 8 after transplantation into B6 mice (gray fill). Open histograms depict background staining on BM-derived CD11b+ cells of normal B6 mice. (**C**) The bar graph shows the mean absolute number (±SD) of BABL.B (H-2^b^, CD45.2)-derived myeloid cells expressing GFP (either control or BCR-ABL1) in B6 (H-2^b^, CD45.1) recipients at day 8 after transplantation (MHC-I matched, unrelated, induced-self recognition).

To address whether NK cell activation by non-self MHC-I conferred a protective effect, we transplanted BCR-ABL1 expressing B6D^d^ BM precursors into B6 recipients. In this donor/recipient combination, host NK cells partially reject normal B6D^d^ BM allografts based on the H-2D^d^-specific Ly49D activation receptor [37]. However, NK cells in B6 recipients failed to impact the expansion of BCR-ABL1+ B6D^d^ myeloid cells (**Fig. 2A**), indicating that NK cell mediated non-self recognition is not effective against primary BCR-ABL1+ cells *in vivo*.

There is considerable evidence that the engagement of the NKG2D activation receptor by stress-induced ligands plays an important role for the recognition of transformed cells by NK cells [20]. While B6-background BCR-ABL1+ myeloid cells do not express the NKG2D ligands Rae-1 (**Fig. 2B**) or Mult1 (**not shown**), myeloid cells from certain mouse strains upregulate Rae-1 following BM transplantation and this results in graft rejection in specific recipient mouse strains [38]. Accordingly, we used the MHC-I matched, unrelated strain combination of BALB.B (H-2^b^)−>B6 (H-2^b^) to address whether induced-self recognition can control BCR-ABL1+ cells. However, despite significant expression of Rae-1 (but not MULT1 or H60) (**Fig. 2B** and **data not shown**) we failed to observe a significant NK cell-mediated effect on BALB.B BCR-ABL1+ myeloid cells (**Fig. 2C**). Of note, though, we observed a significant NK cell-dependent rejection of normal BALB.B myeloid cells (**Fig. 2C**), in agreement with [38]. Thus, despite the expression of NKG2D ligand, induced-self recognition of BCR-ABL1+ cells *in vivo* is impaired.

### NK cell mediated missing-self recognition can protect from CML disease *in vivo*

**Figure 3:**
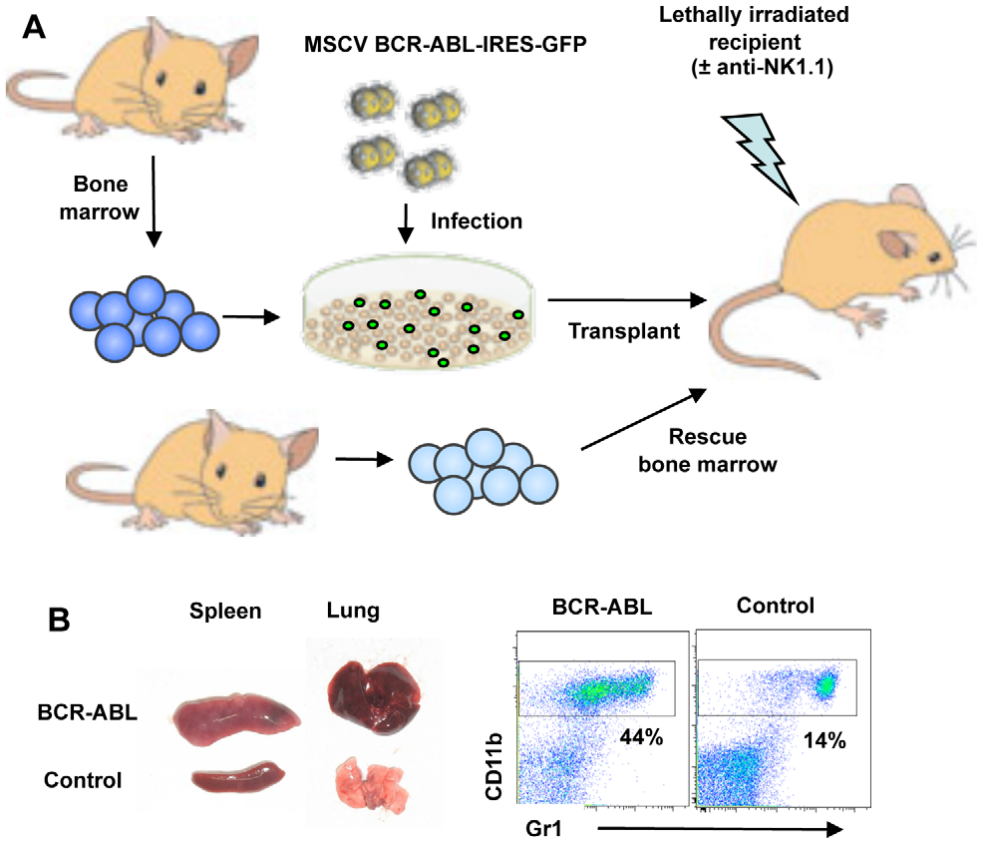
Schematic representation of CML protection assays. (**A**) NK cell rejection of normal BM allografts is lethal between d12 and d14 post transplantation. To ensure the survival of host mice beyond this time point, lethally irradiated recipient mice were transplanted with mixtures of MHC-I matched rescue BM plus MHC-I-different BCR-ABL1 transduced BM. (**B**) Mixed BM grafts rapidly induces CML disease, which is characterized by pulmonary hemorrhage, splenomegaly and increased numbers of mature granulocytes in peripheral blood.

Since NK cell mediated missing-self recognition impacted myeloid expansion at d8 post transplantation, we next determined whether recipient mice were protected from CML disease *in vivo*. Since NK cell rejection of normal BM allografts is lethal between d12 and d14 post transplantation we ensured the long-term survival of host mice by co-transplanting MHC-I matched rescue BM (**Fig. 3A**). Similar to the transplantation with a single type of BM, such mixed BM grafts rapidly induced CML disease, which is characterized by weight loss, increased numbers of peripheral-blood cells (with a predominance of mature granulocytes), splenomegaly and pulmonary hemorrhage, owing to granulocyte infiltration into the lung (**Fig. 3B**) [31].

**Figure 4:**
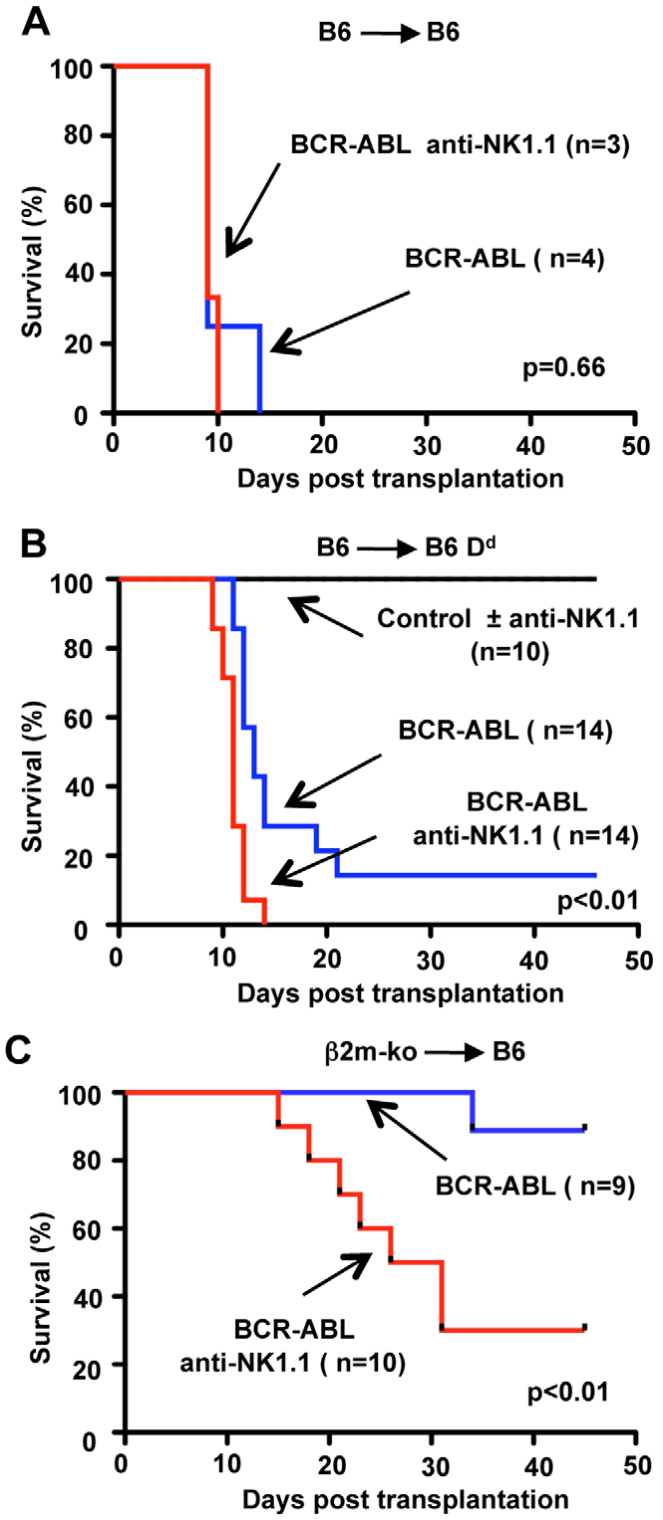
NK cell-mediated missing-self recognition can protect from BCR-ABL1 induced CML disease. **A** Survival graph of B6 recipients (H-2^b^) transplanted with a mixture of BCR-ABL1 transduced B6 BM cells (H-2^b^) and non-transduced B6 rescue BM (H-2^b^). Some recipient mice had been depleted of NK1.1+ cells by the injection of mAb PK136 (anti-NK1.1). These data are derived from a single experiment. **B** Survival graph of B6D^d^ recipients (H-2^b^D^d^) transplanted with mixtures of control transduced B6 BM (H-2^b^) and non-transduced B6D^d^ rescue BM (H-2^b^D^d^). All recipient mice survived independent of the presence or absence of NK1.1+ cells (±anti-NK1.1). Survival of B6D^d^ recipients (H-2^b^D^d^) transplanted with mixtures of BCR-ABL1 transduced B6 BM (H-2^b^) and non-transduced B6D^d^ rescue BM (H-2^b^D^d^). In the presence of NK cells, the survival of recipients is significantly improved (*p*<0.01). The data have been compiled from three independent experiments. **C** Survival graph of B6 recipients (H-2^b^) transplanted with mixtures of BCR-ABL1 transduced β2m-ko (MHC-I^low^) BM and non-transduced B6 rescue BM (H-2^b^). In the presence of NK cells, the survival of recipients is significantly improved (*p*<0.01). The data have been compiled from two independent experiments.

When BCR-ABL1+ BM was transplanted into MHC-I matched recipients, the presence of NK cells did not improve the survival of recipient mice, delay the onset of disease (**Fig. 4A**) or alter any of the symptoms associated with CML disease (**data not shown**). Corresponding observations were made when BCR-ABL1+ B6 BM was transplanted into NK cell-sufficient RAG-1-knock out mice (H-2^b^) and into NK cell-deficient RAG-1 common γ chain double-knock-out mice (**data not shown**). Thus, as expected, NK cells do not impact MHC-I matched CML disease.

The transplantation of B6 BCR-ABL1+ BM into B6D^d^ hosts, from which NK1.1+ cells had been depleted, resulted in CML disease in 100% of recipient mice (mean onset day 11.0±1.3, n = 14) (**Fig. 4B**). The presence of host NK cells significantly delayed disease onset (mean onset day 13.7±3.1, n = 12)(p<0.01). In addition, in the presence of NK cells, only 12 of 14 mice (86%) succumbed to CML disease. The remaining recipients (2/12, 14%) remained disease free (>50d) (**Fig. 4B**). Thus a partial Ly49 ligand mismatch reduced CML disease *in vivo*.

**Figure 5:**
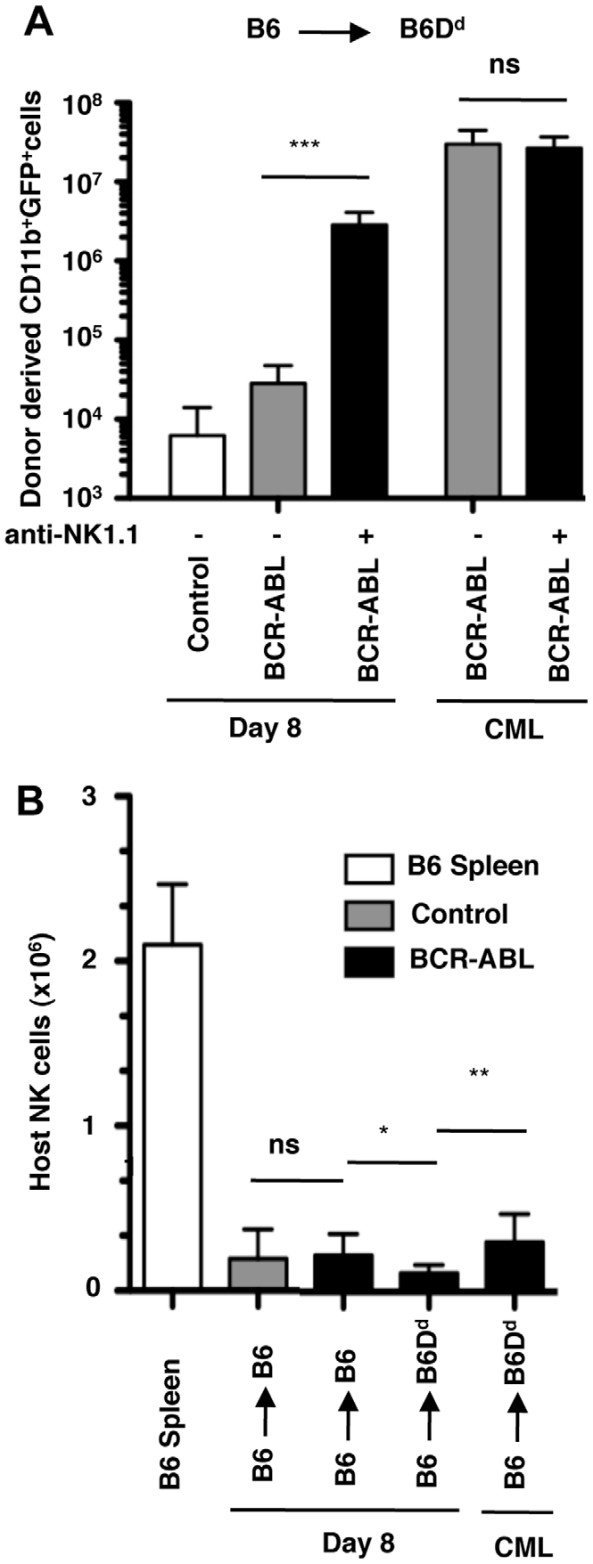
BCR-ABL1+ myeloid cells escape NK cell mediated control. (**A**) The bar graph shows the mean number (±SD) of donor-derived GFP+ (control or BCR-ABL1) myeloid cells in the spleen of recipient mice at d8 after transplantation (left) and when CML disease had developed (right). Some recipient mice had been depleted of NK1.1+ cells by the injection of mAb PK136 (anti-NK1.1). (**B**) Mean number (±SD) of host-derived NK cells in the spleen of recipient mice at d8 after transplantation and when CML disease had developed (right). Significant differences between groups is indicated as * p<0.05, ** p<0.01 and *** p<0.001; (ns) not significantly different p>0.05.

In contrast to the early time point after transplantation (d8) (**Fig. 1C, D**), animals with CML disease had comparable numbers of BCR-ABL1+ myeloid cells, irrespective of whether NK cells were deleted or not (**Fig. 5A**). The eventual failure to prevent disease progression was not due to a loss of NK cells. On the contrary, NK cells were actually slightly more abundant at later time points (**Fig. 5B**). Thus the initial control of leukemic cells is incomplete and BCR-ABL1+ myeloid cells eventually escaped NK cell-mediated missing-self recognition.

Finally we tested whether complete MHC-I mismatch provided an improved protection against CML disease. In the absence of NK cells, the majority of B6 recipients receiving β2m-ko BCR-ABL1+ BM developed disease symptoms (70%, 7/10) (**Fig. 4C**). In contrast, when NK cells were present most recipients of β2m-ko BCR-ABL1+ BM remained disease free (89%, 8/9) for >45d (**Fig. 4C**). Interestingly, the only recipient that developed disease, had symptoms characteristic of B-ALL (B cell acute lymphocytic leukemia) rather than CML. B-ALL is characterized by a moderate splenomegaly and enlarged lymph nodes due to an expansion of B220+ B cells and such mice do not have the extensive infiltration of the lung and liver characteristic of CML (data not shown). In the absence of NK cells, some B6 recipients of BCR-ABL1+ β2m-ko BM also developed B-ALL (2/7 mice that were diseased). However most recipients developed CML (5/7). These experiments formally show that following BM transplantation, NK cell mediated missing-self recognition can protect from CML disease.

### NK cell recognition of BCR-ABL1+ leukemia initiating cells

**Figure 6:**
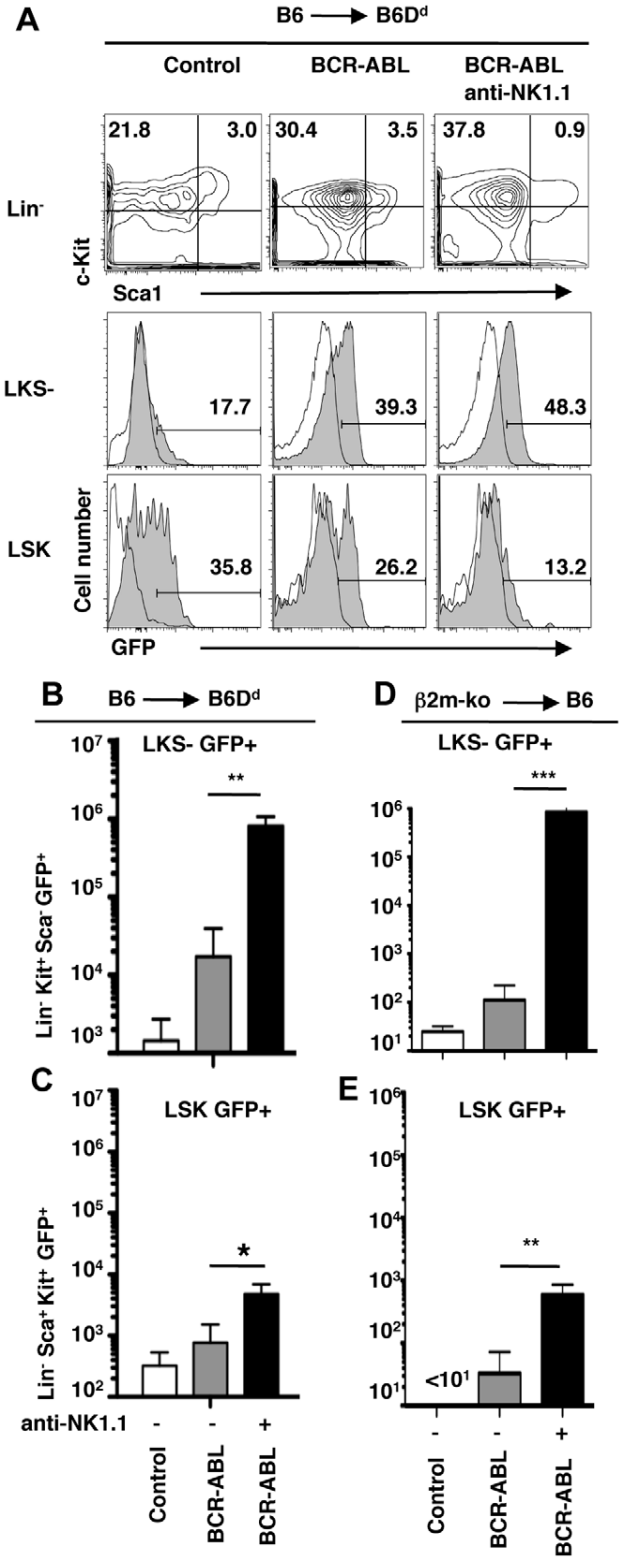
NK cell mediated missing-self recognition reduces the abundance of BCR-ABL1+ leukemia initiating cells. (**A**) B6 BM cells (H-2^b^) were transduced with a retrovirus expressing GFP (Control) or BCR-ABL1 plus GFP (BCR-ABL1) and transplanted into lethally irradiated B6D^d^ recipient mice (H-2^b^D^d^). Recipient spleens were analyzed by flow cytometry at d8 post transplantation. Donor-derived cells lacking markers of mature lineage cells (Lin-) were analyzed for the expression of c-kit and Sca-1 (top row). Histograms (gray filled) depict GFP expression (Control or BCR-ABL1) in gated Lin- c-kit+ sca-1- (LKS-) cells (myeloid/erythroid progenitors) (middle row) and in Lin- c-kit+ sca-1+ (LSK) cells (haematopoietic stem cell compartment) (bottom row). Open histograms depict background staining using BM precursor cells from normal B6 mice. Numbers indicate the percentage of cells in the respective gate. Some recipient mice had been depleted of NK1.1+ cells (anti-NK1.1). The bar graphs show the mean absolute number (±SD) of donor-derived GFP+ (Control or BCR-ABL1) LKS- cells (myeloid/erythroid progenitors) (**B**) and LSK cells (haematopoietic stem cell compartment) (**C**) at d8 after transplantation. (**D, E**) The bar graphs show the mean absolute number (±SD) of β2m-ko-derived GFP+ (Control or BCR-ABL1) LKS- cells (**D**) and LSK cells (**E**) at d8 after transplantation into B6 (H-2^b^) recipients. Significant differences between groups are indicated as * p<0.05, ** p<0.01 and *** p<0.001; (ns) not significantly different p>0.05.

Finally we addressed whether NK cells exerted their protective effect by targeting mature BCR-ABL1+ myeloid cells, immature myeloid precursors or the small population of leukemia initiating cells, which propagate CML disease [39]. As mentioned above, NK cell mediated missing-self recognition strongly reduced the abundance of mature BCR-ABL1+ myeloid cells (**Fig. 1**). Moreover, myeloid/erythroid progenitor cells (Lin- Kit+ Sca-1- (LKS-)) GFP+ were also strongly reduced (**Fig. 6A, B, D**), suggesting that NK cells efficiently target immature myeloid progenitors. Finally, the abundance of leukemia initiating cells, which are present in the haematopoietic stem cell compartment (Lin- Sca-1+ c-kit+) (LSK) expressing BCR-ABL1 [40], was significantly reduced based on a partial MHC-I deficiency of the graft relative to the host (**Fig. 6A, C**). Leukemia initiating cells were essentially absent when the graft lacked MHC-I molecules (**Fig. 6E**). These data show that NK cell-mediated missing-self recognition can target leukemia initiating cancer stem cells and that this correlates with protection from CML disease.

## Discussion

A potential role of NK cells to prevent leukemia relapse has been suggested based on the retrospective analysis of data from patients receiving unrelated HLA matched or partially HLA mismatched bone marrow transplantation. However, direct evidence that NK cells can prevent leukemia relapse is lacking and it has remained unclear what NK cell recognition events might be effective. Here we provide direct evidence that NK cells can be effective against primary CML *in vivo*. Unexpectedly, a protective effect was only observed for NK cell mediated missing-self recognition whereby an increased MHC-I receptor-ligand mismatch improved the protective effect *in vivo*. In contrast, the positive recognition of allogeneic MHC-I exerted no measurable effect on the abundance of BCR-ABL1-expressing myeloid cells *in vivo*. Further, it was possible that lethal irradiation/BM transplantation produced a cytokine milieu that reversed the reduced responsiveness of activation receptors on non-educated NK cells (i.e. Ly49A+ and Ly49G2+ NK cells in H-2^b^ mice), which are not inhibited by the MHC-I of the tumor. However, the absence of an effect of NK cells upon MHC-I matched transplantation provides circumstantial evidence that uneducated NK cells do not become reactive against BCR-ABL1+ cells. Finally, the recognition of stress-induced ligands by NKG2D also failed to mediate a significant protective effect against leukemia, even in a situation where the respective ligand(s) are expressed. Since normal BM cells that express NKG2D ligand are rejected, the data raise the possibility that BCR-ABL1 expression impairs the recognition of cells expressing NKG2D ligand. This defect was not based on a down-regulation or loss of the NKG2D receptor on NK cells (data not shown). Collectively, we have been unable to identify NK cell recognition events that allow the selective detection of BCR-ABL1+ as compared to normal host cells. Rather this study has identified NK cell recognition events that are impaired due to the expression of BCR-ABL1. Missing-self recognition was the only NK cell recognition strategy, which was not impaired and which significantly impacted the course of CML disease.

The NK cell activation receptors (and their ligands) mediating missing-self recognition remain incompletely defined. It is clear, however, that NK cells are activated by multiple receptors specific for constitutively expressed ligands on healthy host cells. The predominant reactivity of NK cells for cells of haematopoietic origin is explained in part by the expression of SLAM family receptors/ligands by haematopoietic cells [41]. Such restricted expression of ligands may also explain the observation that NK cells do not mediate GvHD [42]. The identification of all the activating receptors and their ligands will be required to explain why missing-self recognition is particularly effective against CML and whether the recognition of CML is dependent on the same or partially distinct activation receptors. Moreover, this knowledge will be essential to understand how CML eventually escapes NK cell mediated missing-self recognition. In addition to specific receptor-ligand interactions, it is also possible that the particular cytokine milieu generated in the course of conditioning and BM transplantation favors missing-self reactivity.

Here we have aimed at determining the potential of NK cells to protect from CML following BM transplantation. The advantage of our set-up is that the efficacy of NK cells against BCR-ABL1+ cells can be compared to that against normal cells, which is well established based on classical BM graft rejection experiments. Indeed, as detailed above, we did identify significant differences between the control of normal and BCR-ABL1+ BM progenitors. It is clear, however, that GvL is mediated by donor-derived and not by host-derived NK cells. Thus future studies will verify whether missing-self recognition by donor-derived NK cells is equally effective. While missing-self recognition has the potential to protect from CML disease, the efficacy of host NK cells against partially mismatched CML was limited. Despite a delay in disease onset, the majority of recipient mice (12/14; 86%) eventually succumbed to CML disease. While NK cells largely controlled BCR-ABL1+ myeloid cells at an early time-point after transplantation (day 8), in diseased animals the abundance of CML cells and their precursors was comparable, independent of the presence of NK cells. The eventual lack of control was not due to a complete loss host NK cells. On the contrary, host NK cells were slightly more abundant in the spleen of diseased mice. In agreement with these data, normal allogeneic BM induces the expansion of adoptively transferred NK cells [43]. Irrespective of this, BCR-ABL1+ cells eventually escape NK cell mediated control. The limited efficacy of NK cells may be explained by irradiation damage to host NK cells. Indeed, we have noted that lethal and even sub-lethal irradiation significantly reduces the abundance of splenic NK cells (5 fold). Moreover, within the first few days after transplantation residual host NK cells do not proliferate [44] (and our unpublished data). As donor-derived or adoptively transferred NK cells should not be damaged, it will be of interest to see whether these NK cell populations are more effective against CML. On the other hand, recent data suggest that the systemic exposure of mature NK cells to a large number of MHC-I-deficient normal cells profoundly reduces NK cell function [45]. Induction of NK cell hypo-responsiveness seems to be the outcome when mature NK cells are persistently stimulated under non-inflammatory conditions. Indeed, when NK cells are stimulated in the context of an inflammatory environment their function can stably improve [46], [47]. If so, approaches to keep stimulated NK cells functional in a non-inflammatory environment may be needed to obtain protective effect against leukemia. The mouse models used here should be useful to address these issues.

Significantly we provide evidence that NK cells can impact the course of CML disease by targeting leukemia initiating stem cells that are refractory to the control by BCR-ABL1 tyrosine kinase inhibitors. These data suggest that NK cell recognition has the potential to cure CML. Finally, our findings validate approaches to treat leukemia using mAb-based blockade of self-specific inhibitory MHC-I receptors [48], [49] and they may influence the selection of optimal donor/recipient combinations for stem cell transplantation to treat leukemia.

## Materials and Methods

### Mice

C57BL6 (B6) mice (H-2^b^; CD45.2) were purchased from Harlan Olac (The Netherlands), CD45.1 congenic B6 mice (H-2^b^; CD45.1). BALB.B (H-2^b^; CD45.2) and β2m-deficient B6 mice (MHC-I^low^; CD45.2) were originally purchased from The Jackson lab (Bar Harbor, ME) and maintained at the LICR. H-2D^d^ transgenic mice, backcrossed >10 generation to B6 (B6 D^d^) (H-2^b^D^d^; CD45.2) have been described before [34]. Animal experimentation followed protocols reviewed and approved by the Service Vétérinaire du Canton de Vaud (authorization number 1124).

### Retroviral infection and bone marrow transplantation

Retroviral infection and BM transplantation was performed essentially as described in [31]. Briefly, plasmids containing MSCV IRES GFP and MSCV BCR-ABL1 (p210) IRES GFP were transiently transfected into 293T cells for high titer virus production using standard procedures. BM donor mice were injected with 5-Fluorouracil (5-FU) (0.15 mg/ml) and BM cells were harvested 4 days later. BM cells were cultured in DMEM (plus 10% FCS, 50 mM 2-ME, 50 U/ml penicillin/50 µg/ml streptomycin, 10 mM HEPES) supplemented with IL-3 (10 ng/ml), IL-6 (10 ng/ml) and SCF (50 ng/ml) for 48 h and infected with retrovirus in the presence of polybrene (8 µg/ml). After 24 h, 10^5^ cells (usually around 10% GFP+) were injected i.v. into lethally irradiated recipient mice (2 doses of 480rad 4 h apart from a ^137^Cs source). One day prior to irradiation, some recipient mice were injected i.p. with 200 µg of mAb PK136 (anti-NK1.1) to deplete NK1.1+ cells. Recipient mice were sacrificed at day 8 after transplantation and analyzed by flow cytometry (see below). Alternatively, for leukemia induction experiments, irradiated recipient mice were transplanted with a mixture of infected BM and non-infected (MHC-I-matched) rescue BM (10^5^ each), both derived from 5-FU treated donors. Recipient mice were monitored daily for weight loss and failure to thrive. Pre-morbid animals were sacrificed and relevant tissues were harvested and analyzed visually and by flow cytometry (see below). For continuous depletion of NK1.1+ cells, recipient mice were injected i.p. with 200 µg of mAb PK136 (anti-NK1.1) one day prior to irradiation and mAb injection was repeated every 2 weeks.

### Flow cytometry

Spleen and BM cells were incubated with 2.4G2 (anti-CD16/32) hybridoma supernatant (to block Fc receptors) before staining for multi-color flow cytometry. The following mAbs were used: CD3ε (17A2), CD4 (GK1.5), CD8 (53.6.7), CD11b (Mac1)(M1/70), CD41 (MWReg30), CD45R (B220) (RA3-6B2), CD45.1 (A20.1), CD45.2 (104), CD117 (ACK2), CD127 (A7R34), CD135 (FLT3R)(A2F10.1), CD161 (NK1.1) (PK136), GR1 (RB6-8C5), TCRβ (H57), TCRγδ (GL3) and Ter119, Rae-1 (186107: R&D), Mult-1 (5D10: eBiosciences). Abs were conjugated with appropriate fluorochromes at the LICR or purchased from BD PharMingen (San Diego, CA) or eBioscience (San Diego, CA).

A cocktail of FITC-conjugated mAbs to TCRβ, TCRγδ, CD3ε, CD4, CD8, Mac-1, B220, NK1.1, Ter119 and GR1 was used to gate out cells expressing markers of mature lineage-cells (Lin) cells. Samples were run on a FACSCanto flow cytometer and analyzed with Cell Quest or FACS Diva software (Becton Dickinson, San Jose, CA).

### Statistical analysis

A two-tailed student's t-test was used to determine significant differences between data sets and a Gehan-Wilcoxon test was used to compare survival curves. Data sets were considered significantly different when *p*<0.05.

## References

[1] Shami P, Deiniger M (2011). Evolving treatment strategies for patients newly diagnosed with chronic myeloid leikemia: the role of second-generation BCR-ABL inhibitors as first-line therapy.. Leukemia.

[2] Mahon FX, Réa D, Guilhot J, Guilhot F, Huguet F (2010). Discontinuation of imatinib in patients with chronic myeloid leukaemia who have maintained complete molecular remission for at least 2 years: the prospective, multicentre Stop Imatinib (STIM) trial.. Lancet Oncol.

[3] Gratwohl A, Heim D (2009). Current role of stem cell transplantation in chronic myeloid leukaemia.. Best Pract Res Clin Haematol.

[4] Ruggeri L, Capanni M, Casucci M, Volpi I, Tosti A (1999). Role of natural killer cell alloreactivity in HLA-mismatched hematopoietic stem cell transplantation.. Blood.

[5] Miller JS, Soignier Y, Panoskaltsis-Mortari A, McNearney SA, Yun GH (2005). Successful adoptive transfer and in vivo expansion of human haploidentical NK cells in patients with cancer.. Blood.

[6] Ruggeri L, Mancusi A, Burchielli E, Aversa F, Martelli MF (2007). Natural killer cell alloreactivity in allogeneic hematopoietic transplantation.. Curr Opin Oncol.

[7] Moretta A, Sivori S, Vitale M, Pende D, Morelli L (1995). Existence of both inhibitory (p58) and activatory (p50) receptors for HLA-C molecules in human natural killer cells.. J Exp Med.

[8] Nakamura MC, Linnemeyer PA, Niemi EC, Mason LH, Ortaldo JR (1999). Mouse Ly49D recognizes H-2D^d^ and activates natural killer cell cytotoxicity.. J Exp Med.

[9] Karlhofer FM, Ribaudo RK, Yokoyama WM (1992). MHC class I alloantigen specificity of Ly-49+ IL-2 activated natural killer cells.. Nature.

[10] Colonna M, Samaridis J (1995). Cloning of immunoglobulin-superfamily members associated with HLA-C and HLA-B recognition by human natural killer cells.. Science.

[11] Ljunggren HG, Kärre K (1990). In search of the ‘missing self’: MHC molecules and NK cell recognition.. Immunol Today.

[12] Ohlen C, Kling G, Höglund P, Hansson M, Scangos G (1989). Prevention of allogeneic bone marrow graft rejection by H-2 transgene in donor mice.. Science.

[13] Fernandez NC, Treiner E, Vance RE, Jamieson AM, Lemieux S (2005). A subset of natural killer cells achieve self-tolerance without expressing inhibitory receptors specific for self MHC molecules.. Blood.

[14] Kim S, Poursine-Laurent J, Truscott SM, Lybarger L, Song YJ (2005). Licensing of natural killer cells by host major histocompatibility complex class I molecules.. Nature.

[15] Anfossi N, Andre P, Guia S, Falk CS, Roetynck S (2006). Human NK cell education by inhibitory receptors for MHC class I.. Immunity.

[16] Chalifour A, Scarpellino L, Back J, Brodin P, Devèvre E (2009). A role for cis interaction between the inhibitory Ly49A receptor and MHC class I for NK cell education.. Immunity.

[17] Orr MT, Murphy WJ, Lanier LL (2010). ‘Unlicensed’ natural killer cells dominate the response to cytomegalovirus infection.. Nat Immunol.

[18] Hill GR, Crawford JM, Cooke KR, Brinson YS, Pan L (1997). Total body irradiation and acute graft-versus-host disease: the role of gastrointestinal damage and inflammatory cytokines.. Blood.

[19] Bauer S, Groh V, Steinle A, Phillips JH, Lanier LL (1999). Activation of NK cells and T cells by NKG2D, a receptor for stress-inducible MICA.. Science.

[20] Guerra N, Tan YX, Joncker NT, Choy A, Gallardo F (2008). NKG2D-deficient mice are defective in tumor surveillance in models of spontaneous malignancy.. Immunity.

[21] Davies SM, Ruggieri L, DeFor T, Wagner JE, Weisdorf DJ (2002). Evaluation of KIR ligand incompatibility in mismatched unrelated donor hematopoietic transplants. Killer immunoglobulin-like receptor.. Blood.

[22] Brunstein CG, Wagner JE, Weisdorf DJ, Cooley S, Noreen H (2009). Negative effect of KIR alloreactivity in recipients of umbilical cord blood transplant depends on transplantation conditioning intensity.. Blood.

[23] Cooley S, Trachtenberg E, Bergemann TL, Saeteurn K, Klein J (2009). Donors with group B KIR haplotypes improve relapse-free survival after unrelated hematopoietic cell transplantation for acute myelogenous leukemia.. Blood.

[24] Pende D, Marcenaro S, Falco M, Martini S, Bernardo ME (2009). Anti-leukemia activity of alloreactive NK cells in KIR ligand-mismatched haploidentical HSCT for pediatric patients: evaluation of the functional role of activating KIR and redefinition of inhibitory KIR specificity.. Blood.

[25] Leung W, Iyengar R, Turner V, Lang P, Bader P (2004). Determinants of antileukemia effects of allogeneic NK cells.. J Immunol.

[26] Hsu KC, Gooley T, Malkki M, Pinto-Agnello C, Dupont B (2006). KIR ligands and prediction of relapse after unrelated donor hematopoietic cell transplantation for hematologic malignancy.. Biol Blood Marrow Transplant.

[27] Miller JS, Cooley S, Parham P, Farag SS, Verneris MR (2007). Missing KIR ligands are associated with less relapse and increased graft-versus-host disease (GVHD) following unrelated donor allogeneic HCT.. Blood.

[28] Benjamin JE, Gill S, Negrin RS (2010). Biology and clinical effects of natural killer cells in allogeneic transplantation.. Curr Opin Oncol.

[29] Pegram HJ, Ritchie DS, Smyth MJ, Wiernik A, Prince HM (2011). Alloreactive natural killer cells in hematopoietic stem cell transplantation.. Leuk Res.

[30] Daley GQ, Van Etten RA, Baltimore D (1990). Induction of chronic myelogenous leukemia in mice by the P210bcr/abl gene of the Philadelphia chromosome.. Science.

[31] Pear WS, Miller JP, Xu L, Pui JC, Soffer B (1998). Efficient and rapid induction of a chronic myelogenous leukemia-like myeloproliferative disease in mice receiving P210 bcr/abl-transduced bone marrow.. Blood.

[32] Murphy WJ, Kumar V, Bennett M (1987). Acute rejection of murine bone marrow allografts by natural killer cells and T cells.. J Exp Med.

[33] Öhlén C, Kling G, Höglund P, Hansson M, Scangos G (1989). Prevention of allogeneic bone marrow graft rejection of H-2 transgene in donor mice.. Science.

[34] Ioannidis V, Zimmer J, Beermann F, Held W (2001). Cre recombinase-mediated inactivation of H-2Dd transgene expression: evidence for partial missing-self recognition by Ly49A NK cells.. J Immunol.

[35] Hanke T, Takizawa H, McMahon CW, Busch DH, Pamer EG (1999). Direct assessment of MHC class I binding by seven Ly49 inhibitory NK cell receptors.. Immunity.

[36] Brodin P, Lakshmikanth T, Johansson S, Karre K, Hoglund P (2009). The strength of inhibitory input during education quantitatively tunes the functional responsiveness of individual natural killer cells.. Blood.

[37] Raziuddin A, Longo AL, Mason L, Ortaldo JR, Bennett M (1998). Differential effects of the rejection of bone marrow allografts by the depletion of activating versus inhibiting Ly-49 natural killer cell subsets.. J Immunol.

[38] Ogasawara K, Benjamin J, Takaki R, Phillips JH, Lanier LL (2005). Function of NKG2D in natural killer cell-mediated rejection of mouse bone marrow grafts.. Nat Immunol.

[39] Hu Y, Liu Y, Pelletier S, Buchdunger E, Warmuth M (2004). Requirement of Src kinases Lyn, Hck and Fgr for BCR-ABL1-induced B-lymphoblastic leukemia but not chronic myeloid leukemia.. Nat Genet.

[40] Hu Y, Swerdlow S, Duffy TM, Weinmann R, Lee FY (2006). Targeting multiple kinase pathways in leukemic progenitors and stem cells is essential for improved treatment of Ph+ leukemia in mice.. Proc Natl Acad Sci U S A.

[41] Dong Z, Cruz-Munoz ME, Zhong MC, Chen R, Latour S (2009). Essential function for SAP family adaptors in the surveillance of hematopoietic cells by natural killer cells.. Nat Immunol.

[42] Ruggeri L, Capanni M, Urbani E, Perruccio K, Shlomchik WD (2002). Effectiveness of donor natural killer cell alloreactivity in mismatched hematopoietic transplants.. Science.

[43] Olson JA, Zeiser R, Beilhack A, Goldman JJ, Negrin RS (2009). Tissue-specific homing and expansion of donor NK cells in allogeneic bone marrow transplantation.. J Immunol.

[44] Prlic M, Kamimura D, Bevan MJ (2007). Rapid generation of a functional NK-cell compartment.. Blood.

[45] Joncker NT, Shifrin N, Delebecque F, Raulet DH (2010). Mature natural killer cells reset their responsiveness when exposed to an altered MHC environment.. J Exp Med.

[46] Sun JC, Beilke JN, Lanier LL (2009). Adaptive immune features of natural killer cells.. Nature.

[47] Cooper MA, Elliott JM, Keyel PA, Yang L, Carrero JA (2009). Cytokine-induced memory-like natural killer cells.. Proc Natl Acad Sci U S A.

[48] Sola C, Andre P, Lemmers C, Fuseri N, Bonnafous C (2009). Genetic and antibody-mediated reprogramming of natural killer cell missing-self recognition in vivo.. Proc Natl Acad Sci U S A.

[49] Vahlne G, Lindholm K, Meier A, Wickstrom S, Lakshmikanth T (2010). In vivo tumor cell rejection induced by NK cell inhibitory receptor blockade: maintained tolerance to normal cells even in the presence of IL-2.. Eur J Immunol.

